# Recombinant prion protein vaccination of transgenic elk PrP mice and reindeer overcomes self-tolerance and protects mice against chronic wasting disease

**DOI:** 10.1074/jbc.RA118.004810

**Published:** 2018-11-05

**Authors:** Dalia H. Abdelaziz, Simrika Thapa, Jenna Brandon, Justine Maybee, Lauren Vankuppeveld, Robert McCorkell, Hermann M. Schätzl

**Affiliations:** From the ‡ Department of Comparative Biology and Experimental Medicine and; the ¶ Calgary Prion Research Unit, University of Calgary, Calgary, Alberta T2N 4Z6, Canada and; the § Department of Biochemistry and Molecular Biology, Faculty of Pharmacy, Helwan University, Cairo 11795, Egypt

**Keywords:** prion, prion disease, vaccine, neurodegeneration, transgenic mice, bovine spongiform encephalopathy, cervid prion protein, chronic wasting disease, Creutzfeldt-Jakob disease, transgenic elk mice

## Abstract

Chronic wasting disease (CWD) is a fatal neurodegenerative disease that affects cervids in North America and now Europe. No effective measures are available to control CWD. We hypothesized that active vaccination with homologous and aggregation-prone recombinant prion protein (PrP) could overcome self-tolerance and induce autoantibody production against the cellular isoform of PrP (PrP^C^), which would be protective against CWD infection from peripheral routes. Five groups of transgenic mice expressing elk PrP (TgElk) were vaccinated with either the adjuvant CpG alone or one of four recombinant PrP immunogens: deer dimer (Ddi); deer monomer (Dmo); mouse dimer (Mdi); and mouse monomer (Mmo). Mice were then challenged intraperitoneally with elk CWD prions. All vaccinated mice developed ELISA-detectable antibody titers against PrP. Importantly, all four vaccinated groups survived longer than the control group, with the Mmo-immunized group exhibiting 60% prolongation of mean survival time compared with the control group (183 *versus* 114 days post-inoculation). We tested for prion infection in brain and spleen of all clinically sick mice. Notably, the attack rate was 100% as revealed by positive CWD signals in all tested tissues when assessed with Western blotting, real-time quaking-induced conversion, and immunohistochemistry. Our pilot study in reindeer indicated appreciable humoral immune responses to Mdi and Ddi immunogens, and the post-immune sera from the Ddi-vaccinated reindeer mitigated CWD propagation in a cell culture model (CWD-RK13). Taken together, our study provides very promising vaccine candidates against CWD, but further studies in cervids are required to investigate vaccine efficacy in the natural CWD hosts.

## Introduction

Prion diseases are fatal transmissible spongiform encephalopathies in human and animals characterized by distinctive spongiform appearance and neuronal loss in the brain. These diseases are caused by accumulation of the pathological isoform (PrP^Sc^)^5^ of the cellular prion protein (PrP^C^) (1–3). Chronic wasting disease (CWD) is considered the most contagious prion disease and affects both free-ranging and farmed cervids (deer, elk, moose, and reindeer) (4–7). First described and reported to be a prion disease in the United States (8–10), until now it has been detected in North America, South Korea, and recently in Norway and Finland (11–13). The substantial shedding of CWD prion infectivity via urine, feces, and saliva into the environment and prion resistance for many years are driving forces for CWD transmission (14–16). Because of the horizontal transmission nature of the disease via mucosal/oral route and the occurrence in wildlife, the control of disease spread is extremely challenging. Moreover, the potential of zoonotic transmission into humans is an alarming issue and is still an open question (17, 18). Yet, studies have shown its transmissibility into nonhuman primates (squirrel monkeys), both by the intracerebral and oral route (19, 20). There are no preventive or therapeutic measures available against CWD, nor for other prion diseases such as Creutzfeldt-Jakob disease in humans, scrapie in sheep, and bovine spongiform encephalopathy in cattle.

The concept of active and passive immunization has already been introduced for prion disease, and reduced prion propagation *in vitro* and *in vivo*, and it prolonged the incubation period in murine-adapted scrapie prion models after immunization (21–26). Vaccination in prion disease would be useful to prevent peripheral infection before prions reach the brain, as the vast majority of antibodies cannot cross the blood–brain barrier (25–27). Of note, targeting cellular PrP in active immunization is complicated by the necessity to overcome self-tolerance against PrP and by the risk to induce the undesirable side effects. However, there is already a proof-of-concept that active immunization can break the self-tolerance against host prion protein to produce auto-antibodies, without inducing side effects (21, 28, 29). For CWD, there is a very limited number of studies investigating active immunization. A study has reported that active vaccination with synthetic peptides against CWD was not successful in terms of protection in mule deer (30). However, another study reported partial protection against orally challenged CWD infection (around 25%) provided by oral vaccination of white-tailed deer with attenuated *Salmonella typhimurium* vaccine expressing cervid PrP (31). A recent study described a potential CWD vaccine consisting of a nonreplicating human adenovirus that expresses a truncated rabies glycoprotein G fused with postulated disease-specific epitopes, named the rigid loop region (hAd5:tgG-RL). This vaccine was successful in inducing humoral immune responses, both systemic and mucosal, upon oral immunization of white-tailed deer (32).

Our objective in this study was to develop a CWD vaccine that overcomes self-tolerance and induces self-antibodies against cervid prion protein to impede peripheral prion infection. For this purpose, we used multimeric and aggregation-prone recombinant PrPs (both mouse and deer), as our lab had already provided a proof-of-principle that this approach can induce a robust humoral immunity against PrP^C^, both mouse and cervid (21, 28, 29), and protect some immunized mice against scrapie challenge (23). In this study, we tested these recombinant immunogens for their potential to induce immune responses in transgenic mice expressing elk PrP (TgElk) and in reindeer, and we then studied the vaccination effect in TgElk mice against CWD challenge.

## Results

### Immunization of TgElk mice with mouse or deer recombinant PrP induces anti-PrP antibodies

In this vaccination study, we used TgElk mice as a mouse model for CWD. These mice are homozygous for elk PrP, with a 2.5-fold higher expression of PrP^C^ in the brain compared with WT mice (33). An advantage of this mouse model is the very short incubation period (90–110 days) following intracerebral (i.c.) inoculation compared with most other CWD mouse models (33, 34), which may exceed 250 days (35).

**Figure 1.**
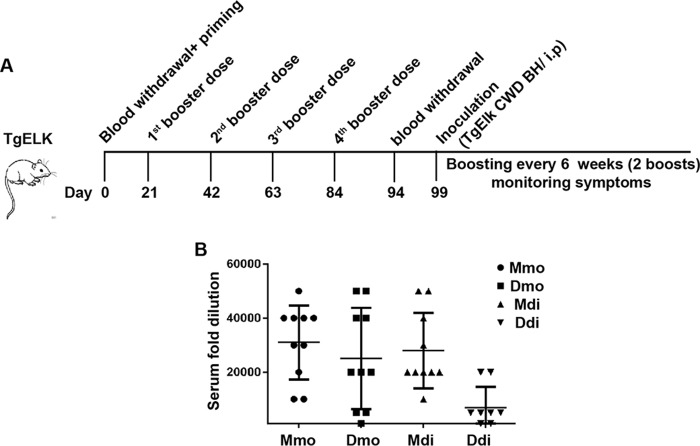
**High-antibody titers produced by immunization of TgElk mice with monomeric or dimeric recombinant PrP.** *A,* TgElk mice were immunized with four different immunogens at 3-week intervals five times (one priming and four booster doses), and blood sampling was performed either before starting vaccination or 10 days after the fourth booster dose. The animals were i.p. inoculated at day 99 with 1% brain homogenate (*BH*) from a terminally ill CWD-infected TgElk mouse, and the animals received two more booster doses post-inoculation at 6-week intervals. *B,* antibody titers using end-point ELISA from the four vaccinated groups. Mice were vaccinated with Mmo, Dmo, Mdi, or Ddi recombinant PrPs, and CpG was used as adjuvant for all groups. The antibody titer for each individual mouse was determined by end-point dilution. The *y* axis indicates the serum fold dilution. The cutoff was calculated as three times average OD (405 nm) of preimmune sera.

In our vaccination study, we used mouse and deer recombinant PrP immunogens in both the monomeric and dimeric form. The structure of the immunogens has been described extensively in our previous work (21, 28, 29). Type B CpG oligonucleotide (CpG) was used as adjuvant based on previous data that indicate that using CpG as adjuvant was efficient in breaking self-tolerance against PrP. All mice were subjected to one priming dose (100 μg of protein) and four boosting doses (50 μg of protein) applied subcutaneously, with 3-week intervals, before inoculating them with elk CWD prions via the intraperitoneal (i.p.) route (Fig. 1*A*). We kept boosting the mice every 6 weeks (two boosts) after challenging them.

Testing the reactivity of the post-immune sera against deer recombinant PrP using ELISA showed high antibody titers in all vaccinated groups (Fig. 1*B*). The lowest titers were found in the Ddi-vaccinated group. The Ddi immunogen is more a self-antigen than is the respective mouse PrP-derived immunogen (Mdi), indicating better efficiency of nonself-immunogens in breaking self-tolerance against cellular PrP in this model. The post-immune sera detected elk PrP (total PrP (−PK) and PrP^Sc^ (+PK)) in immunoblot, with sera from the Mmo-vaccinated group showing the best detection (data not shown).

Taken together, all four immunogens used in our study could successfully break self-tolerance for PrP in TgElk mice and produce high-antibody titers reactive against recombinant and authentic cervid PrP.

### Vaccination of TgElk mice prolonged their survival after CWD challenge

The high-antibody titers in the post-immune sera of all vaccinated groups encouraged us to investigate the protective effect of these antibodies against CWD challenge *in vivo*. For this purpose, we challenged the immunized mice with elk CWD. The rationale for our vaccine approach is interfering in peripheral prion replication, before the process of neuroinvasion is completed. This aligns with the fact that antibodies are not crossing the blood–brain barrier under normal conditions (36). Consequently, this prompted us to use the i.p. route for inoculating the mice, a peripheral route of infection that simulates the natural infection situation better than the i.c. infection.

**Figure 2.**
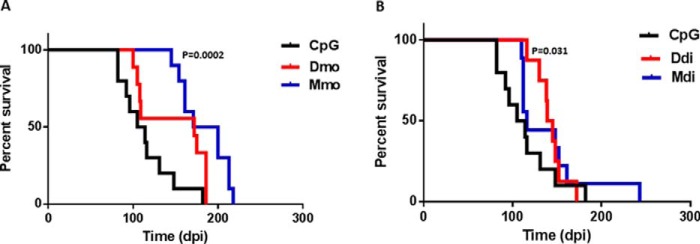
**Immunization with monomeric or dimeric recombinant PrPs prolongs the survival in a CWD-infected TgElk mouse model.** *A* and *B,* Kaplan-Meier survival curves of Dmo, Mmo, Ddi, and Mdi-vaccinated groups compared with control group (CpG-only) of female TgElk mice, inoculated via i.p. route with 1% BH of a terminally ill CWD-infected TgElk mouse. *A,* CpG control group, *n* = 10, median incubation time 109 days post-inoculation (*dpi*). Dmo-vaccinated group, *n* = 9, median incubation time 172 dpi (*p* = 0.0509; log rank test). Mmo-vaccinated group, *n* = 10, median incubation time 185 dpi (*p* = 0.0002; log rank test). *B,* CpG control group, *n* = 10, median incubation time 109 dpi. Ddi-vaccinated group, *n* = 8, median incubation time 142 dpi (*p* = 0.031; log rank test). Mdi-vaccinated group, *n* = 10, median incubation time 116 dpi (*p* = 0.198; log rank test).

After the fourth booster dose, all vaccinated and control TgElk mice were inoculated intraperitoneally with 1% brain homogenate (BH) from terminally ill TgElk mice infected with elk CWD. The mean survival times are displayed in Table 1. All vaccinated groups displayed markedly improved survival times compared with the adjuvant-only group (CpG). The Mmo-vaccinated group exhibited 60% prolongation in their mean survival time compared with the CpG control group (Fig. 2*A*), which is statistically significant (*p* = 0.0002). The Dmo- and Ddi-vaccinated groups showed 28.4 and 24.1% prolongation, respectively, in mean survival time compared with the CpG group (Fig. 2, *A* and *B*). The extension of survival time for the Ddi-vaccinated group was statistically significant (*p* = 0.031) compared with the control group; however, the survival of the Dmo group was not significant when compared with the CpG group (*p* = 0.0509). The Mdi group showed only a 15.9% prolongation, which was not statistically significant (*p* = 0.198). Notably, all inoculated mice showed progressive clinical symptoms, including severe weight loss, kyphosis, very slow movement, ataxia, disorientation, and dragging the hind limbs.

**Table 1:** **Mean survival time and attack rate of the TGElk vaccinated groups** The following abbreviation is used: days post-CWD inoculation.

Vaccination groups	Attack rate	Survival time (dpi) (mean ± S.E.)
CpG	10/10	114.8 ± 10.0
Mmo	10/10	183.6 ± 8.8
Mdi	10/10	133.1 ± 15.0
Dmo	9/9	147.4 ± 13.4
Ddi	8/8	142.5 ± 5.8

In summary, all vaccinated TgElk mice showed prolongation in the survival after CWD challenge compared with the control group, and this prolongation reached 60% in the Mmo-vaccinated group. However, once the mice reached the terminal stage, mice from all groups displayed very comparable clinical signs.

### Intraperitoneally infected mice show PK-resistant PrP^CWD^ signature similar to the inoculum

**Figure 3.**
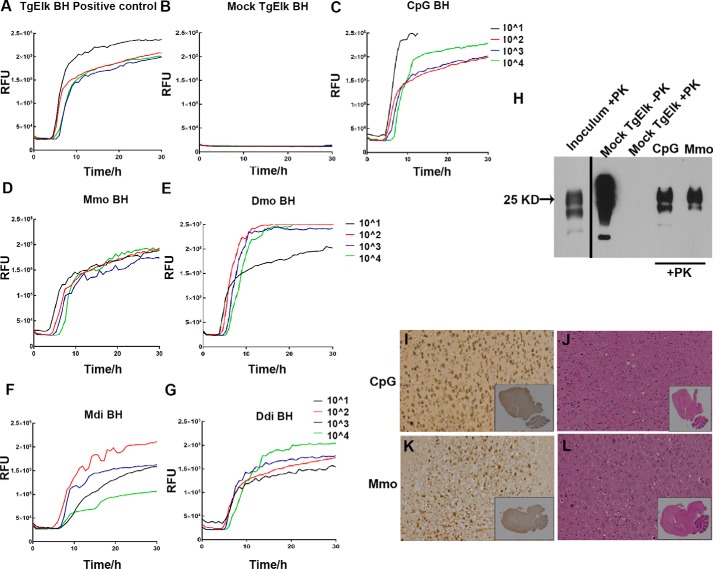
**Prion conversion activity and PrP^Sc^ in brain homogenates of i.p.-inoculated TgElk mice.** *A,* CWD-positive control for RT-QuIC in which the seed was 10% BH of i.c.-inoculated TgElk mouse (terminally ill) after NaPTA enrichment. *B,* brain homogenate of an uninfected TgElk mouse purified using NaPTA was used as a negative control. *C–G,* RT-QuIC assay of one representative brain homogenate from every group upon NaPTA enrichment CpG-only (*C*); Mmo-immunized (*D*); Dmo-immunized (*E*); Mdi-immunized (*F*), and Ddi-immunized (*G*) groups. Mouse rPrP was used as substrate for all reactions, four 10-fold dilutions of seed were analyzed (10^−1^ to 10^−4^), and each curve was plotted as average of four technical replicates against time. *H,* Western blot analysis using mAb 8H4 of brain homogenate samples (CpG and Mmo-vaccinated mice) treated with PK followed by NaPTA enrichment. The inoculum is brain homogenate from a terminally ill TgElk mouse which was i.c.-infected with elk CWD. Mock-infected TgElk brain homogenate was used as a negative control. *I* and *K,* immunohistochemistry for PrP^Sc^ using BAR224 antibody in representative brain sections from CpG control group (*I*) and Mmo-vaccinated group (*K*). The sections showed positive staining in different areas of the brain. High magnification (×20) areas are shown by *boxes* in whole hemisphere sagittal section inlet. *J* and *L,* brain sections were stained with hematoxylin and eosin to detect spongiform pathological changes in sections from one representative CpG control (*J*) and one representative Mmo-vaccinated brain (*L*).

To the best of our knowledge, studies reporting the intraperitoneal infection of TgElk mice are lacking. This led us to assess the infection in the brain of the terminally sick mice using various readouts to confirm the complete clinical attack rate. The brain homogenates from all terminally sick TgElk mice demonstrated prion conversion activity as shown by RT-QuIC assay (Fig. 3, *C–G*). The mock brain homogenate from the uninfected TgElk mouse was used as negative control, and brain homogenate of intracerebrally CWD-infected and terminally sick TgElk mouse was used as a positive control. Notably, the crude brain homogenate from the vaccinated and intraperitoneally infected TgElk mice in our study initially showed only weak positive signals in RT-QuIC assay (Fig. S1), which was strongly increased after sodium phosphotungstic acid purification (Fig. 3 and Fig. S1). Moreover, immunoblot analysis of PK-digested brain homogenates from i.p.-infected TgElk mice demonstrated a PrP^Sc^ signature comparable with the CWD inoculum PrP^Sc^ pattern (Fig. 3*H*). Of note, spleen homogenates isolated from all the CWD clinically sick TgElk mice showed remarkable conversion activity in the RT-QuIC assay, which was comparable with the spleen homogenates isolated from i.c.-infected TgElk mice, even without sodium phosphotungstic acid enrichment (Fig. S2, *A–G*). In addition, PK-digested spleen homogenates from i.p.-infected TgElk mice showed PK-resistant PrP in immunoblot (Fig. S2*H*). This denotes substantial amounts of infectivity in the spleen of the i.p.-infected TgElk mice.

For further confirmation of complete attack rate in i.p.-infected TgElk mice, we performed immunohistochemistry (IHC) staining for PrP^Sc^ in brain sections from both vaccinated and unvaccinated mice (CpG-only). The IHC images of both Mmo-vaccinated and CpG-control mouse brain sections showed positive staining in the brain with PrP^Sc^ aggregates found mainly in prefrontal cortex, temporal lobes, and occipital lobe (Fig. 3, *I* and *K*). H&E staining of the brain sections showed spongiform pathological changes that were comparable in both vaccinated and nonvaccinated mice (Fig. 3, *J* and *L*). Given that all the mice were sacrificed at the terminal stage, we could not detect any characteristic differences in the distribution of PrP^Sc^ deposits in their brains.

Taken together, we demonstrate clear signs of prion infection in brains of i.p.-infected TgElk mice by RT-QuIC, immunoblot, and IHC analysis.

### Vaccination of reindeer with multimeric immunogens induces auto-antibodies

**Figure 4.**
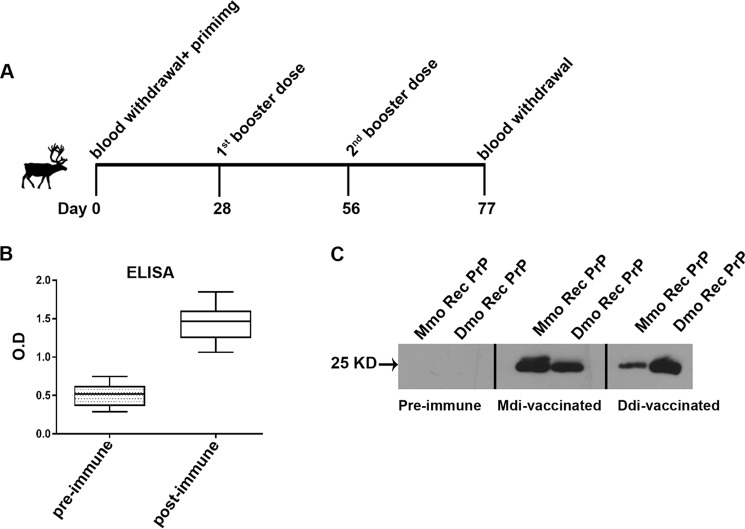
**Immunization of reindeer with dimeric recombinant PrP induces anti-PrP antibodies.** *A,* seven reindeer were vaccinated three times with either Mdi (four animals) or Ddi (three animals) immunogens at 4-week intervals (one priming and two booster doses), and blood sampling was performed either before starting vaccination (preimmune) or 21 days after the second booster dose (post-immune). *B,* ELISA for post-immune sera of the Mdi-vaccinated reindeer (1:100 dilution shown), compared with the respective preimmune sera. The *y* axis represents the optical density (405 nm). *C,* both mouse and deer monomeric recombinant PrP were subjected to immunoblot analysis. As primary antibodies, the preimmune sera (reindeer ID: 15S) and post-immune sera of reindeer vaccinated with Mdi (reindeer ID: 15S) or Ddi (reindeer ID: 3WD) immunogens with comparable post-immune antibody titers, all in 1:500 dilution, were used.

The successful vaccination of TgElk mice with recombinant PrP immunogens prompted us to assess the immunogenicity of the dimeric recombinant PrPs in reindeer, as a relevant large-animal model. The *Prnp* genotypes of the reindeer used in this study are displayed in Table 2. Seven reindeer were vaccinated with either Mdi (*n* = 4) or Ddi (*n* = 3). They were immunized subcutaneously with 500 μg of protein/animal, and CpG was used as an adjuvant. Each reindeer received three doses with 4-week intervals, and blood samples were drawn before the priming dose and 3 weeks after the last booster dose (Fig. 4*A*). PrP-specific antibody titers were determined in sera of the vaccinated reindeer using PrP-coated ELISA. All Mdi-immunized reindeer showed significant humoral immune response to the recombinant PrP (Fig. 4*B*). However, only one of the three reindeer responded to the Ddi vaccination with detectable ELISA titers (Table 2). Interestingly, the responding animal was 225Y and 176D polymorphic, which may play a role in the response to the self-Ddi immunogen as it creates some differences between self-PrP and Ddi immunogen, which was not the case for the other two WT reindeer.

**Table 2:** **Vaccinated reindeer genotypes and response to immunogens** The following abbreviations are used: NR, nonresponder; Y, tyrosine; N, asparagine; D, aspartic acid. The antibody titers for individual deer were determined by end-point dilution. The cutoff was calculated as three times average OD (405 nm) of pre-immune sera.

Deer ID	Genotype	Immunogen	Antibodies titer (serum fold dilution)
2S	WT	Mdi	1:5000
15S	225Y	Mdi	1:100
3X	225Y + 138 N	Mdi	1:1000
2Y	225Y	Mdi	1:1000
3WD	225Y + 176D	Ddi	1:100
3YD	WT	Ddi	NR
5XD	WT	Ddi	NR

End-point ELISA titers were higher in Mdi-vaccinated animals (up to 1/5000 as shown in Table 2) compared with Ddi-vaccinated reindeer that had low or nondetectable titers. This indicates that the nonself Mdi immunogen was more able under these vaccine conditions to induce a humoral immune response against PrP as tested in ELISA than the self-Ddi immunogen. Yet, Ddi post-immune sera showed better detection of Dmo recombinant PrP in immunoblots (Fig. 4*C*).

We were not able to test the protective efficacy of immunization as challenging of reindeer with CWD was not feasible at our facilities. Of note, we did not observe any unwanted side effects in the vaccinated reindeer.

In summary, vaccination of reindeer with mouse dimeric recombinant PrP immunogen was able to overcome self-tolerance to PrP in this relevant large animal model.

### Antibodies from Ddi-immunized reindeer diminish prion propagation in CWD-infected RK13 cells

**Figure 5.**
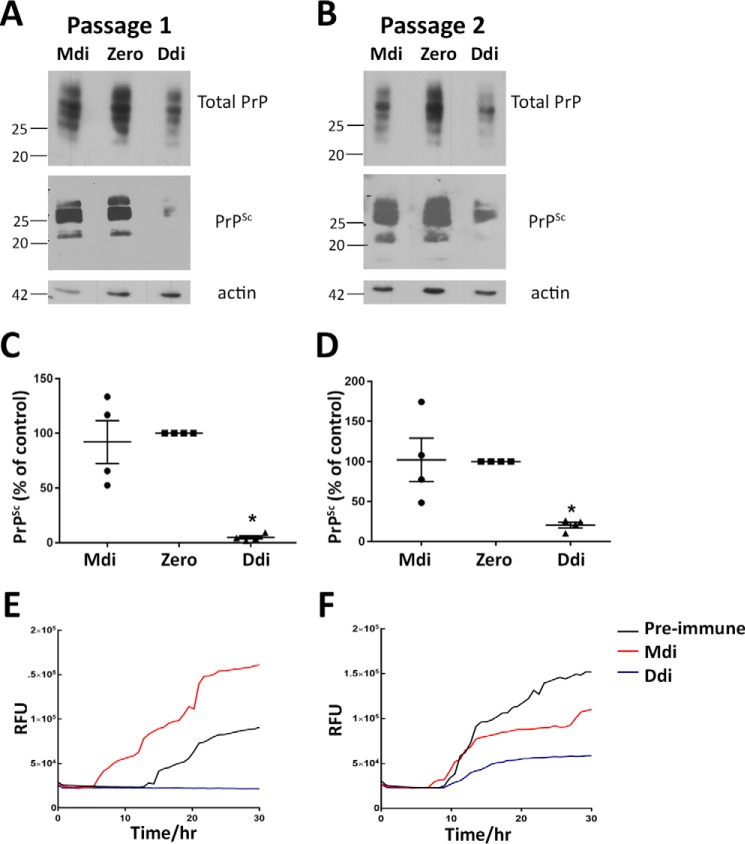
**Post-immune sera of Ddi-immunized reindeer reduce PrP^Sc^ propagation in CWD-infected RK13 cells.** *A* and *B,* CWD-infected RK13 cells expressing cervid PrP^C^ (CWD RK13) were treated with post-immune sera with comparable titers from reindeer vaccinated with Mdi (reindeer ID: 15S) or Ddi (reindeer ID 3WD), while passaging the cells every 7 days for two passages. Treatment with preimmune sera (here denoted as zero time sera) from reindeer ID: 15S was used as control. Sera were used at a dilution of 1:100 in culture media. Cells from every passage were lysed and subjected to PK digestion (+*PK*) or not (−*PK*). The lysates were immunoblotted with mAb 8H4, and actin was used as loading control. *C* and *D,* densitometric analysis of immunoblots from treated CWD RK13 cells. Data are represented as percentage of control (preimmune sera) and normalized with amount of actin. *, *p* < 0.05 is considered significant. *E* and *F,* RT-QuIC assay was performed on passage 1 (*E*) and 2 (*F*) of CWD-RK13 cells treated with post-immune sera from reindeer vaccinated with Mdi or Ddi. Treatment with preimmune sera (zero) was done for comparison. Post-nuclear lysates were used as seeds and mouse recombinant PrP as substrate for RT-QuIC. The seeding activity at the 10^−4^ dilution was used for comparative analysis (shown here). The *y* axis shows relative ThT fluorescence units (*RFU)*; the *x* axis represents time in hours.

To test the efficiency of reindeer post-immune sera in inhibiting CWD propagation, we treated persistently CWD-infected RK13 cells (overexpressing cervid PrP) with post-immune antibodies from Ddi- or Mdi-vaccinated reindeer, using preimmune sera as control. Treatment of CWD-infected RK13 cells with the Ddi post-immune sera significantly reduced PrP^Sc^ as shown by quantitative immunoblot analysis, whereas Mdi sera were not effective (Fig. 5, *A–D*). RT-QuIC assay confirmed these results as the seeding activity of the Ddi post-immune sera–treated cell lysates was strongly decreased compared with preimmune sera or Mdi sera–treated cells (Fig. 5, *E* and *F*). This indicates that the self-antibodies formed against reindeer PrP by using Ddi immunogen are more efficient in binding to native and authentic PrP^C^ and suppressing CWD prion propagation than the cross-reactive antibodies induced by Mdi immunogen.

In summary, these data show that self-antibodies induced in vaccinated reindeer have the potential to interfere in cellular CWD prion propagation.

## Discussion

Chronic wasting disease is a highly contagious and fatal neurodegenerative disease of cervids, which is found in North America and Korea and, more recently, in Europe. CWD affects farmed and free-ranging animals, and environmental components play a major role in disease transmission. This makes CWD very hard to control, and the disease is spreading geographically and in numbers in many parts of Northern America. It was shown that a high incidence of CWD negatively affects the population survival (37, 38). Although the zoonotic potential of CWD is currently considered low, data in nonhuman primates are conflicting for macaque transmissions (20, 39, 40), and infection by the oral route was clearly shown for new-world monkeys. A further alarming factor is that CWD prions are coming in different shapes and can undergo mutation–selection-like processes (41–43). No effective therapies or vaccines are currently available to control CWD. The causative agent is the pathological and misfolded form of the endogenous host cellular prion protein (PrP^Sc^), which does not elicit any detectable adaptive immune response due to having the same primary sequence as PrP^C^. This makes developing an effective vaccine against CWD very challenging, and most approaches aim for overcoming the self-tolerance to PrP. However, such an approach involves the risk of inducing unwanted side effects, and therefore, careful control of auto-immunity side effects have to be in place (44).

Only a few studies reported on the protective effect of a vaccination approach against CWD infection (30–32). Most of them were unsuccessful in achieving protection except one study by Goni *et al.* (31), which reported 25% protection in a white-tailed deer infection model, in the form of prolongation of incubation time to disease. This study therefore provides a proof-of-concept for efficacy of a vaccination approach for containing CWD (31). This highlights the importance of our current study that describes a promising vaccination approach aimed at containing a fatal and so far uncontrollable disease. In our previous work (21, 23, 28, 29), we provided a proof-of-concept in WT and transgenic mouse models for the efficacy of mouse and deer recombinant PrPs, in both monomeric and dimeric forms, to break self-tolerance and to induce self-antibodies against cellular PrP. Of note, post-immune sera were effective in inhibiting prion propagation in persistently prion-infected cell culture models, including CWD-infected RK13 cells (21, 29).

In this study, we extended our vaccination approach to transgenic mice expressing elk PrP (TgElk mice), which we challenged with elk CWD using the intraperitoneal route. All four immunogens induced again a substantial humoral immune response when testing for anti-PrP antibodies in ELISA. Mouse monomeric and dimeric recombinant PrPs showed the highest titers in ELISA, which can be explained by the nonself (mouse) nature of these immunogens. Despite the high sequence homology between mouse and deer PrP, there are more than 20 different amino acids between them that may help provoke an adaptive immune response. The salient finding of our study is prolongation of survival in all groups of vaccinated TgElk mice after intraperitoneal CWD challenge, compared with CpG adjuvant-only control mice. Although our previous studies with mouse PrP in WT mice identified only a fraction of mice with prolongation of survival time (23), effects in this study pointed toward a group effect, although with some variability. The group vaccinated with Mmo immunogen displayed around 60% prolongation in mean survival, which was the highest among all vaccinated groups. In line with this, sera of Mmo-vaccinated mice demonstrated the best detection of total elk PrP in immunoblots (data not shown). However, mice vaccinated with Mdi immunogen had only a 15.9% prolongation of mean survival time, although these sera were very reactive in ELISA and immunoblot. Obviously and in line with our previous results (21, 28, 29), there is no clear correlation with reactivity in ELISA and immunoblot and in protective behavior in prion-infected cell culture assays and mouse models. The groups immunized with Dmo and Ddi immunogens demonstrated extended mean survival times of 28.4% and 24.1% respectively. Interestingly, Ddi sera showed the weakest reactivity in ELISA. Somewhat unexpectedly, there was no difference among groups in terms of reactivity in the linear epitope mapping (data not shown).

One important beneficial aspect of CWD vaccination should be mitigation of peripheral shedding from infected animals. This aligns well with our experimental strategy, which wants to block prion propagation at peripheral sites of the body, outside the brain. We have started to monitor prion shedding in CWD-infected TgElk mice using RT-QuIC analysis from urine samples. Detection of prion infectivity in such excreta of infected TgElk mice has been challenging so far due to the limited sample size and the fact that such transgenic mice do not well recapitulate prion pathogenesis and prion shedding as is characteristic for cervids. TgElk mice are homozygous for the elk PrP transgene, and they can propagate CWD prions from deer and elk origins. Moreover, compared with other transgenic cervidized mouse models, TgElk mice are characterized by a very short incubation time (95–120 days) upon CWD infection (33, 34). However, in previous studies using the TgElk mouse model infection was always done using the intracerebral route. Because our antibody approach targets PrP^C^ as substrate of prion propagation outside the central nervous system, we had to use the intraperitoneal infection route to test the efficiency of our vaccination approach. In addition, intraperitoneal infection resembles the natural routes of infection, which usually occur through the alimentary tract. In our previous study, we had i.p.-infected Tg(cerPrP)1536^+/+^ mice with cervid prions, which resulted in an incomplete attack rate (data not shown), which makes our data inconclusive. To the best of our knowledge, this study is the first that characterizes i.p. infection in TgElk mice. All inoculated mice demonstrated complete attack rate as shown by typical clinical signs, ranging from weight loss, kyphosis, very slow movement, rough coat, to ataxia. These symptoms were in accordance with signs reported by Jeon *et al.* (34). in this mouse line. The average incubation time for the control nonvaccinated group was 114 days, which is longer than the i.c. infection (90–95 days) reported in previous studies (33, 34). This is expected, as i.p. infection is usually longer in incubation time than i.c. inoculation. Of note, due to the vaccination schedule, our mice were 14–15 weeks older than i.c.-injected mice, which are usually between 4 and 6 weeks old when infected with prions. This might explain the broad range in incubation time.

To verify complete attack rate, we investigated the brains of all i.p.-infected mice for signs of CWD infection using three different readouts. RT-QuIC is a versatile technique for diagnosis of prion diseases, including CWD (45). Although crude brain homogenates from all i.p.-inoculated mice were positive in RT-QuIC, their signals were usually weaker than the ones of i.c.-infected brain homogenates of the same mouse line. After NaPTA enrichment of PrP^Sc^, all brain homogenates from i.p.-infected mice showed strong signals comparable with that of i.c.-infected mice in both RT-QuIC and immunoblotting. In contrast, seeding activity in RT-QuIC of spleen homogenates of i.p.-infected mice was as high as found in i.c.-infected spleen homogenates. Histologically, PrP^Sc^ deposition and vacuolation were detected with a pattern reminiscent of the pathological changes reported by LaFauci and colleagues in i.c.-inoculated TgElk mice. Whether there is a difference in the propagation of CWD in central *versus* peripheral infection requires further analysis.

In our pilot study in reindeer, all Mdi-vaccinated reindeer showed appreciable antibody titers in ELISA. Yet, the response to Ddi as immunogen was only 33% (1 of 3) in ELISA. The responding animal had two *Prnp* polymorphisms (225Y and 176D) that could explain the humoral immune response. However, better response to Ddi immunogen may be achievable by optimizing dose and timing of vaccination. So far, we used only one dose (500 μg/animal), and the schedule was not intense. Moreover, it is normal to find variation in vaccine responsiveness in a given population, including some nonresponders. Of note, we will do a recall experiment for the vaccinated reindeer with two more boosts after 1 year, to find out whether we are looking at nonresponse or a low response. Despite the comparatively low titer in ELISA, Ddi but not Mdi post-immune sera from reindeer were effective in neutralizing prion propagation in persistently CWD-infected RK13 cells. This indicates that superior binding to authentic and native cervid PrP^C^ is achieved by self-antibodies in Ddi post-immune sera (Fig. 4*C*). In line with this, Mdi post-immune sera efficiently bound to mouse PrP but less efficiently to deer PrP when used as primary antibody in immunoblots. This comes in accordance with our previous data in which post-immune sera from Dmo-vaccinated tg(cerPrP)1536^+/+^ mice significantly reduced PrP^Sc^ propagation in CWD-infected RK13 cells. Our cell culture results indicate an improved efficiency of self-antibodies over cross-reactive antibodies in binding to native and authentic PrP^C^, which provides protection against conversion into PrP^Sc^. However, in this study, we were not able to use Mmo and Dmo as immunogens due to the limited number of available reindeer for our study. We are aware of the fact that parenteral routes can be used for farmed animals and that the oral route is more realistic for vaccination of wild animals. Thus, we started encapsulating our immunogens in polylactic–co-glycolic acid microspheres that can be used orally (46). As an alternative approach, we also have initiated attempts to express PrP in plant-based systems.

In conclusion, our study provides very promising vaccine candidates against CWD, which were able to prolong the incubation time to disease by 60% in a transgenic mouse model. In addition, our pilot study in reindeer provided evidence for feasibility and efficacy of our vaccine approach in cervids. However, further studies are required to investigate the protective effect of the vaccine in cervids that are the natural hosts of chronic wasting disease.

## Experimental procedures

### Ethics statement

The animal use in the experiments strictly followed the guidelines of the Canadian Council for Animal Care. The animal protocol was approved by the institutional Health Sciences Animal Care Committee. For vaccination of mice, the protocol was under number AC14-0122, and for reindeer it was AC17-0088.

### Reagents and antibodies

PK and Pefabloc (PK inhibitor) were commercially obtained from (Roche Applied Science, Germany). Primary antibodies used were as follows: anti-PrP mAb 4H11 (47) anti-PrP mAb 8H4 (Sigma), anti-PrP mAb BAR224 (Cayman Chemical), and anti-β-actin (Sigma). As secondary peroxidase-conjugated antibodies, goat anti-mouse HRP (Jackson ImmunoResearch) and rabbit anti-deer HRP (Kirkegaard & Perry Laboratories) were used.

### Mice

The transgenic mouse line used in this study (TgElk mice) was kindly provided by Dr. Debbie McKenzie, Centre for Prions and Protein Folding Diseases, University of Alberta, Edmonton, Canada. The development of transgenic mice, genotyping, maintenance of homozygous line for the Rocky Mountain elk (*Cervus elaphus nelsoni*) PrP and their susceptibility to CWD prions have been described previously (33). In our facility, the transgenic line was maintained on a homozygous background, and only female mice were used in this study.

### Immunogen preparation

The preparation of immunogens is described elsewhere (29). Briefly, pQE30 (Qiagen) expression vector was used to express the monomeric and dimeric constructs in *Escherichia coli* strain BL21-Gold(DE3) pLysS (Stratagene) as described previously (21, 28). Nickel-nitrilotriacetic acid Superflow resin beads (Qiagen) were used to separate His-tagged immunogens from the bacterial lysate. After elution and refolding of protein by dialysis, the concentration of protein was determined using the BCA kit (Pierce, ThermoFisher Scientific, Rockford, IL).

### Mouse vaccination and prion challenge studies

The female TgElk mice used in the study were immunized via subcutaneous route by injecting 100 μg of protein/mouse, starting with 4–6-week-old mice. After this first priming dose, each mouse was subjected to four booster doses of 50 μg, each within 3-week intervals. The oligodeoxynucleotide CpG (5 μm) (InvivoGen, San Diego) was used as an adjuvant for all immunogens, and the control group received only CpG. The bleeding schedule for all mice was maintained twice, before immunization (zero sera), and 10 days after the last boost (post-immune sera). Five days after the post-immune bleeding, all mice were inoculated with mouse-adapted Elk CWD prions by injecting 100 μl of 1% BH i.p. The inoculum was obtained from terminal prion-sick TgElk mice inoculated with elk CWD intracerebrally. For the preparation of BH, mouse brains were homogenized in PBS at a final concentration of 10% (w/v) and stored at −80 °C. Two booster doses were given to each mouse after prion inoculation at 6-week intervals. The mice were monitored daily after start of first disease signs. Two researchers were involved in monitoring animals, recording and scoring clinical signs, and making decision for euthanasia of animals. The weight of animals was also recorded every day. Brain and spleen samples were collected from terminally sick mice; 10% homogenate was prepared using PBS and kept at −80 °C until use. Half of the brain from each animal was kept in formalin for histology examination.

### Reindeer immunization study

Seven reindeer (*Rangifer tarandus*) accommodated in the Veterinary Sciences Research Station at the University of Calgary Spy Hill Campus were vaccinated three times with either Mdi (four animals) or Ddi (three animals) immunogens with 4-week intervals (one priming and two booster doses). The animals were immunized with 500 μg of Mdi or Ddi with 5 μm CpG added as adjuvant via subcutaneous route with animals being restrained in chutes. Animals and injection sites were monitored for any adverse effects of immunization. Blood sampling was performed either before starting vaccination (preimmune) or 21 days after the second booster dose (post-immune).

### ELISA

ELISA was done following the procedure described previously (29). Briefly, 1 μg of recombinant protein in sodium-carbonate buffer (pH 9.5) was used to coat the wells of high-binding 96-well plates (Greiner Bio-One GmbH-Frickenhausen-Germany) for overnight. Blocking was done using 3% bovine serum albumin (BSA) in PBST for 2 h at 37 °C after washing with PBST. A serial dilution of sera in 3% BSA was prepared, and plates were incubated with sera for 1 h. Following the washing step, either HRP-labeled anti-mouse IgG antibody (Jackson ImmunoResearch, West Grove, PA) or rabbit anti-deer HRP (Kirkegaard & Perry Laboratories) was added as secondary antibody. 2,2′-Azino-bis(3-ethylbenzothiazoline-6-sulfonic acid) peroxidase (Kirkegaard & Perry Laboratories) was used as substrate for signal detection, and OD was measured at 405 nm using a BioTek Synergy HT reader.

### Cell culture experiments and PK digestion

Cervid PrP-expressing RK13 cells (cerRK13) were kindly obtained from Dr. Glenn Telling's lab (Colorado State University, Fort Collins). Dulbecco's modified Eagle's media (Gibco) with 10% fetal bovine serum, 1% penicillin/streptomycin, 1 μg/ml puromycin, and 200 μg/ml G148 was used for maintenance of cerRK13 cells. To boost infection, cells were infected again with 10% brain homogenate from white-tailed deer CWD-infected terminally sick Tg (cerPrP) 1536 ^+/+^ mice, and this persistently infected CWD-cell culture model was used for cell experiments. Cells were cultured in medium with a 1:100 dilution of sera from Mdi- or Ddi-vaccinated reindeer, and preimmune sera were used as control. After 5 days, cells were lysed in cold lysis buffer (10 mm Tris-HCl, pH 7.5; 100 mm NaCl; 10 mm EDTA; 0.5% Triton X-100; 0.5% sodium deoxycholate), and lysates were digested with 20 μg/ml PK at 37 °C for 30 min. PK digestion was blocked using 0.5 mm Pefabloc, and samples were subjected to methanol precipitation. Precipitated proteins were dissolved in TNE buffer (50 mm Tris-HCl, pH 7.5; 150 mm NaCl; 5 mm EDTA).

### NaPTA precipitation

BH or spleen homogenate (SH) was prepared in PBS using a gentle MACs Dissociator (Miltenyi Biotech) in a 10% w/v dilution. For immunoblotting and RT-QuIC, 250 and 50 μl, respectively, of BH or SH was mixed with Sarkosyl to make the final concentration to 2% and incubated by shaking at 37 °C for 30 min. For immunoblotting, BH was digested with 40 μg/ml PK at 37 °C for 30 min before adding Sarkosyl; for SH, 50 μg/ml PK was used for 1 h. Then, 0.5 mm Pefabloc was added to stop PK digestion. After incubation in Sarkosyl, 1/12.5 volume of NaPTA stock solution (20 mm phosphotungstic acid; 400 mm MgCl_2_; 200 mm NaOH; pH 7.4) was added and incubated at 37 °C for 2 h by shaking. Following centrifugation at 21,000 × *g* for 30 min at 8 °C, the pellet was washed with cell lysis buffer (10 mm Tris-HCl, pH 7.5; 100 mm NaCl; 10 mm EDTA; 0.5% Triton X-100; 0.5% sodium deoxycholate) containing 1% Sarkosyl. The solution was again centrifuged for 15 min, and the pellet was either dissolved in RT-QuIC seed dilution buffer for RT-QuIC or in sample buffer for immunoblotting.

### Immunoblotting

Immunoblot analysis was performed as mentioned in our previous studies (29). Briefly, protein precipitated from cell lysate, BH, or SH after NaPTA precipitation was separated on 12.5% SDS-PAGE. The electroblotting was done on Amersham Biosciences Hybond P 0.45 polyvinylidene difluoride membranes (Amersham Biosciences) and analyzed using Luminata Western Chemiluminescent HRP substrates (Millipore). ImageJ software was used to perform densitometric analysis.

### RT-QuIC

RT-QuIC was performed as described previously, including the procedure for preparation of recombinant prion protein using a bacterial expression system (15, 29). Briefly, master mix was prepared with 20 mm sodium phosphate, pH 7.4, 300 mm NaCl, 1 mm EDTA, 10 μm thioflavin T, and 0.1 mg/ml mouse rPrP substrate, from which 98 μl was taken in each well of a black-walled 96-well optical bottom plate (Nalge Nunc International, Nunc). Either BH after NaPTA precipitation, SH without NaPTA precipitation, or cell lysates were used at 10-fold serial dilutions in seed dilution buffers. Reactions included 2 μl of seed in each dilution and were set up in quadruplicate. After sealing with Nunc Amplification Tape (Nalge Nunc International), plates were incubated in a FLUOstar Omega (BMG Labtech, Cary, NC) plate reader for 30 h at 42 °C at 1-min shaking (700 revolutions per min) and 1-min rest cycle. Finally, fluorescence measurements (450 nm excitation and 480 nm emission) obtained every 15 min were averaged from four replicate wells and plotted against reaction time. A positive and a negative control were run on each plate. A reaction cutoff was calculated as average fluorescence of the negative controls plus five times the S.D. of the negatives. Reactions were considered positive when ≥2 replicates of four were reactive (>cut-off).

### IHC

The immunohistochemical analysis of brain sections was kindly performed by the Histology Core Facility at the Centre for Prions and Protein Folding Diseases, University of Alberta, following the procedure as described previously (48). Briefly, formalin-fixed and paraffin-embedded brain tissues were sectioned (4.5–6 μm thick) to get sagittal sections on colorfrost plus slide (ThermoFisher Scientific) and left overnight at 37 °C. The slides were used for immunostaining and H&E staining to investigate the deposition of PrP^Sc^ (CWD) and spongiform changes, respectively. Anti-PrP mAb BAR224 (Cayman Chemical) was used for PrP^Sc^ staining at a dilution of 1:2000. Brain slides were deparaffinized and hydrated using xylene and ethanol dip cycles (100–70% ethanol) and then heated at autoclaving temperature (121 °C) and pressure in 10 mm citric acid buffer (6.0) for 30 min. The slides were cooled at room temperature and incubated in 98% formic acid for 30 min followed by treatment with 4 m guanidine thiocyanate for 2 h at room temperature. 3% hydrogen peroxide was used to remove endogenous peroxidase activity in the tissue. Biotinylation of antibody was done using a kit (DAKO ARK^TM^, ARK animal research kit) following the manufacturer's protocol, and slides were incubated in antibody for overnight at 4 °C in a humidifying chamber. Immunostaining detection was done using a streptavidin-peroxidase system (Invitrogen) and 3,3′-diaminobenzidine (Pharmingen) for enzymatic activity detection. Tissue sections were incubated in Mayers hematoxylin (ThermoFisher Scientific) for counterstaining and scanned using a NanoZoomer 2.0RS scanner (Hamamatsu Photonics, Japan). NanoZoomer digital pathology software (Hamamatsu Photonics, Japan) was used for image processing.

### Statistical analysis

Statistical analysis was done using GraphPad Prism (Graph Pad software Inc., version 7.03.) using nonparametric Kruskal-Wallis test for multiple comparisons among groups. Statistical significance for the immunoblots was expressed as (mean ± S.E.). The percent survival was plotted in Kaplan-Meier plot, and log-rank (Mantel-Cox) test was performed for statistically significant difference between groups. *, *p* ≤ 0.05; **, *p* ≤ 0.01 considered significant.

## Author contributions

D. H. A., S. T., and H. M. S. data curation; D. H. A., S. T., J. B., J. M., L. V., and R. M. formal analysis; D. H. A., S. T., J. B., J. M., and H. M. S. validation; D. H. A., S. T., J. B., J. M., L. V., and R. M. investigation; D. H. A. and S. T. visualization; D. H. A., S. T., J. B., J. M., and H. M. S. methodology; D. H. A., S. T., and H. M. S. writing-original draft; D. H. A., S. T., and H. M. S. project administration; D. H. A., S. T., and H. M. S. writing-review and editing; R. M. and H. M. S. supervision; H. M. S. conceptualization; H. M. S. resources; H. M. S. funding acquisition.

## Supplementary Material

Supporting Information

## Footnotes

5 The abbreviations used are:

PrP^Sc^: scrapie isoform of the prion protein
PrP^C^: cellular isoform of the prion protein
CpG: 5′-cytosine-phosphate-guanine-3′
CWD: chronic wasting disease
PrP: recombinant prion protein
Tg: transgenic
Ddi: deer dimer
Dmo: deer monomer
Mdi: mouse dimer
Mmo: mouse monomer
PK: proteinase K
BH: brain homogenate
SH: spleen homogenate
HRP: horseradish peroxidase
NaPTA: sodium phosphotungstic acid
IHC: immunohistochemistry
RT-QuIC: real-time–quaking-induced conversion assay
H&E: hematoxylin and eosin
dpi: days post-inoculation.

## References

[1] Prusiner S. B. (1982) Novel proteinaceous infectious particles cause scrapie. Science 216, 136–144 10.1126/science.6801762 6801762

[2] Prusiner S. B. (1998) Prions. Proc. Natl. Acad. Sci. U.S.A. 95, 13363–13383 10.1073/pnas.95.23.13363 9811807 PMC33918

[3] Aguzzi A., Sigurdson C., and Heikenwaelder M. (2008) Molecular mechanisms of prion pathogenesis. Annu. Rev. Pathol. 3, 11–40 10.1146/annurev.pathmechdis.3.121806.154326 18233951

[4] Sigurdson C. J., and Aguzzi A. (2007) Chronic wasting disease. Biochim. Biophys. Acta 1772, 610–618 10.1016/j.bbadis.2006.10.010 17223321 PMC2680674

[5] Gilch S., Chitoor N., Taguchi Y., Stuart M., Jewell J. E., and Schätzl H. M. (2011) Chronic wasting disease. Top. Curr. Chem. 305, 51–77 10.1007/128_2011_159 21598099

[6] Saunders S. E., Bartelt-Hunt S. L., and Bartz J. C. (2012) Occurrence, transmission, and zoonotic potential of chronic wasting disease. Emerg. Infect. Dis. 18, 369–376 10.3201/eid1803.110685 22377159 PMC3309570

[7] Mitchell G. B., Sigurdson C. J., O'Rourke K. I., Algire J., Harrington N. P., Walther I., Spraker T. R., and Balachandran A. (2012) Experimental oral transmission of chronic wasting disease to reindeer (*Rangifer tarandus tarandus*). PLoS ONE 7, e39055 10.1371/journal.pone.0039055 22723928 PMC3377593

[8] Williams E. S. (2005) Chronic wasting disease. Vet. Pathol. 42, 530–549 10.1354/vp.42-5-530 16145200

[9] Williams E. S., and Young S. (1980) Chronic wasting disease of captive mule deer: a spongiform encephalopathy. J. Wildl. Dis. 16, 89–98 10.7589/0090-3558-16.1.89 7373730

[10] Williams E. S., and Young S. (1982) Spongiform encephalopathy of Rocky Mountain elk. J. Wildl. Dis. 18, 465–471 10.7589/0090-3558-18.4.465 7154220

[11] Argue C. K., Ribble C., Lees V. W., McLane J., and Balachandran A. (2007) Epidemiology of an outbreak of chronic wasting disease on elk farms in Saskatchewan. Can. Vet. J. 48, 1241–1248 18189044 PMC2081988

[12] Kim T.-Y., Shon H.-J., Joo Y.-S., Mun U.-K., Kang K.-S., and Lee Y.-S. (2005) Additional cases of chronic wasting disease in imported deer in Korea. J. Vet. Med. Sci. 67, 753–759 10.1292/jvms.67.753 16141661

[13] Benestad S. L., Mitchell G., Simmons M., Ytrehus B., and Vikøren T. (2016) First case of chronic wasting disease in Europe in a Norwegian free-ranging reindeer. Vet. Res. 47, 88 10.1186/s13567-016-0375-4 27641251 PMC5024462

[14] Mathiason C. K., Powers J. G., Dahmes S. J., Osborn D. A., Miller K. V., Warren R. J., Mason G. L., Hays S. A., Hayes-Klug J., Seelig D. M., Wild M. A., Wolfe L. L., Spraker T. R., Miller M. W., Sigurdson C. J., et al (2006) Infectious prions in the saliva and blood of deer with chronic wasting disease. Science 314, 133–136 10.1126/science.1132661 17023660

[15] John T. R., Schätzl H. M., and Gilch S. (2013) Early detection of chronic wasting disease prions in urine of presymptomatic deer by real-time quaking-induced conversion assay. Prion 7, 253–258 10.4161/pri.24430 23764839 PMC3783112

[16] Pulford B., Spraker T. R., Wyckoff A. C., Meyerett C., Bender H., Ferguson A., Wyatt B., Lockwood K., Powers J., Telling G. C., Wild M. A., and Zabel M. D. (2012) Detection of PrPCWD in feces from naturally exposed Rocky Mountain elk (*Cervus elaphus nelsoni*) using protein misfolding cyclic amplification. J. Wildl. Dis. 48, 425–434 10.7589/0090-3558-48.2.425 22493117

[17] Waddell L., Greig J., Mascarenhas M., Otten A., Corrin T., and Hierlihy K. (2018) Current evidence on the transmissibility of chronic wasting disease prions to humans–A systematic review. Transbound. Emerg. Dis. 65, 37–49 10.1111/tbed.12612 28139079

[18] Haley N. J., and Hoover E. A. (2015) Chronic wasting disease of cervids: current knowledge and future perspectives. Annu. Rev. Anim. Biosci. 3, 305–325 10.1146/annurev-animal-022114-111001 25387112

[19] Marsh R. F., Kincaid A. E., Bessen R. A., and Bartz J. C. (2005) Interspecies transmission of chronic wasting disease prions to squirrel monkeys (*Saimiri sciureus*). J. Virol. 79, 13794–13796 10.1128/JVI.79.21.13794-13796.2005 16227298 PMC1262585

[20] Race B., Meade-White K. D., Miller M. W., Barbian K. D., Rubenstein R., LaFauci G., Cervenakova L., Favara C., Gardner D., Long D., Parnell M., Striebel J., Priola S. A., Ward A., Williams E. S., et al (2009) Susceptibilities of nonhuman primates to chronic wasting disease. Emerg. Infect. Dis. 15, 1366–1376 10.3201/eid1509.090253 19788803 PMC2819871

[21] Gilch S., Wopfner F., Renner-Müller I., Kremmer E., Bauer C., Wolf E., Brem G., Groschup M. H., and Schätzl H. M. (2003) Polyclonal anti-PrP auto-antibodies induced with dimeric PrP interfere efficiently with PrP^Sc^ propagation in prion-infected cells. J. Biol. Chem. 278, 18524–18531 10.1074/jbc.M210723200 12637572

[22] Schwarz A., Krätke O., Burwinkel M., Riemer C., Schultz J., Henklein P., Bamme T., and Baier M. (2003) Immunisation with a synthetic prion protein-derived peptide prolongs survival times of mice orally exposed to the scrapie agent. Neurosci. Lett. 350, 187–189 10.1016/S0304-3940(03)00907-8 14550926

[23] Polymenidou M., Heppner F. L., Pellicioli E. C., Urich E., Miele G., Braun N., Wopfner F., Schätzl H. M., Becher B., and Aguzzi A. (2004) Humoral immune response to native eukaryotic prion protein correlates with anti-prion protection. Proc. Natl. Acad. Sci. U.S.A. 101, Suppl. 2, 14670–14676 10.1073/pnas.0404772101 15292505 PMC521983

[24] Sigurdsson E. M., Sy M.-S., Li R., Scholtzova H., Kascsak R. J., Kascsak R., Carp R., Meeker H. C., Frangione B., and Wisniewski T. (2003) Anti-prion antibodies for prophylaxis following prion exposure in mice. Neurosci. Lett. 336, 185–187 10.1016/S0304-3940(02)01192-8 12505623

[25] White A. R., Enever P., Tayebi M., Mushens R., Linehan J., Brandner S., Anstee D., Collinge J., and Hawke S. (2003) Monoclonal antibodies inhibit prion replication and delay the development of prion disease. Nature 422, 80–83 10.1038/nature01457 12621436

[26] Heppner F. L., Musahl C., Arrighi I., Klein M. A., Rülicke T., Oesch B., Zinkernagel R. M., Kalinke U., and Aguzzi A. (2001) Prevention of scrapie pathogenesis by transgenic expression of anti-prion protein antibodies. Science 294, 178–182 10.1126/science.1063093 11546838

[27] Wisniewski T., and Goñi F. (2010) Immunomodulation for prion and prion-related diseases. Expert Rev. Vaccines 9, 1441–1452 10.1586/erv.10.131 21105779 PMC3036951

[28] Kaiser-Schulz G., Heit A., Quintanilla-Martinez L., Hammerschmidt F., Hess S., Jennen L., Rezaei H., Wagner H., and Schätzl H. M. (2007) Polylactide-coglycolide microspheres co-encapsulating recombinant tandem prion protein with CpG-oligonucleotide break self-tolerance to prion protein in wild-type mice and induce CD4 and CD8 T cell responses. J. Immunol. 179, 2797–2807 10.4049/jimmunol.179.5.2797 17709493

[29] Abdelaziz D. H., Thapa S., Abdulrahman B., Lu L., Jain S., and Schatzl H. M. (2017) Immunization of cervidized transgenic mice with multimeric deer prion protein induces self-antibodies that antagonize chronic wasting disease infectivity *in vitro*. Sci. Rep. 7, 10538 10.1038/s41598-017-11235-8 28874781 PMC5585258

[30] Pilon J. L., Rhyan J. C., Wolfe L. L., Davis T. R., McCollum M. P., O'Rourke K. I., Spraker T. R., VerCauteren K. C., Miller M. W., Gidlewski T., Nichols T. A., Miller L. A., and Nol P. (2013) Immunization with a synthetic peptide vaccine fails to protect mule deer (*Odocoileus hemionus*) from chronic wasting disease. J. Wildl. Dis. 49, 694–698 10.7589/2012-07-200 23778624

[31] Goñi F., Mathiason C. K., Yim L., Wong K., Hayes-Klug J., Nalls A., Peyser D., Estevez V., Denkers N., Xu J., Osborn D. A., Miller K. V., Warren R. J., Brown D. R., Chabalgoity J. A., et al (2015) Mucosal immunization with an attenuated *Salmonella* vaccine partially protects white-tailed deer from chronic wasting disease. Vaccine 33, 726–733 10.1016/j.vaccine.2014.11.035 25539804 PMC4304998

[32] Taschuk R., Scruten E., Woodbury M., Cashman N., Potter A., Griebel P., Tikoo S. K., and Napper S. (2017) Induction of PrPSc-specific systemic and mucosal immune responses in white-tailed deer with an oral vaccine for chronic wasting disease. Prion 11, 368–380 10.1080/19336896.2017.1367083 28968152 PMC5639826

[33] LaFauci G., Carp R. I., Meeker H. C., Ye X., Kim J. I., Natelli M., Cedeno M., Petersen R. B., Kascsak R., and Rubenstein R. (2006) Passage of chronic wasting disease prion into transgenic mice expressing Rocky Mountain elk (*Cervus elaphus nelsoni*) PrPC. J. Gen. Virol. 87, 3773–3780 10.1099/vir.0.82137-0 17098997

[34] Jeon Y.-C., Choi J.-K., Choi E.-K., Carp R. I., and Kim Y.-S. (2013) Pathological characterization of TgElk mice injected with brain homogenate from elk with chronic wasting disease. J. Vet. Sci. 14, 21–26 10.4142/jvs.2013.14.1.21 23388435 PMC3615228

[35] Hannaoui S., Amidian S., Cheng Y. C., Duque Velásquez C., Dorosh L., Law S., Telling G., Stepanova M., McKenzie D., Wille H., and Gilch S. (2017) Destabilizing polymorphism in cervid prion protein hydrophobic core determines prion conformation and conversion efficiency. PLoS Pathog. 13, e1006553 10.1371/journal.ppat.1006553 28800624 PMC5568445

[36] Klyubin I., Nicoll A. J., Khalili-Shirazi A., Farmer M., Canning S., Mably A., Linehan J., Brown A., Wakeling M., Brandner S., Walsh D. M., Rowan M. J., and Collinge J. (2014) Peripheral administration of a humanized anti-PrP antibody blocks Alzheimer's disease Aβ synaptotoxicity. J. Neurosci. 34, 6140–6145 10.1523/JNEUROSCI.3526-13.2014 24790184 PMC4004804

[37] DeVivo M. T., Edmunds D. R., Kauffman M. J., Schumaker B. A., Binfet J., Kreeger T. J., Richards B. J., Schätzl H. M., and Cornish T. E. (2017) Endemic chronic wasting disease causes mule deer population decline in Wyoming. PLoS ONE 12, e0186512 10.1371/journal.pone.0186512 29049389 PMC5648191

[38] Edmunds D. R., Kauffman M. J., Schumaker B. A., Lindzey F. G., Cook W. E., Kreeger T. J., Grogan R. G., and Cornish T. E. (2016) Chronic wasting disease drives population decline of white-tailed deer. PLoS ONE 11, e0161127 10.1371/journal.pone.0161127 27575545 PMC5004924

[39] Race B., Williams K., Orrú C. D., Hughson A. G., Lubke L., and Chesebro B. (2018) Lack of transmission of chronic wasting disease to cynomolgus macaques. J. Virol. 2018, JVI.00550–18 10.1128/JVI.00550-18 29695429 PMC6026755

[40] Race B., Meade-White K. D., Phillips K., Striebel J., Race R., and Chesebro B. (2014) Chronic wasting disease agents in nonhuman primates. Emerg. Infect. Dis. 20, 833–837 10.3201/eid2005.130778 24751215 PMC4012792

[41] Herbst A., Velásquez C. D., Triscott E., Aiken J. M., and McKenzie D. (2017) Chronic wasting disease prion strain emergence and host range expansion. Emerg. Infect. Dis. 23, 1598–1600 10.3201/eid2309.161474 28820384 PMC5572867

[42] Bian J., Kang H.-E., and Telling G. C. (2014) Quinacrine promotes replication and conformational mutation of chronic wasting disease prions. Proc. Natl. Acad. Sci. U.S.A. 111, 6028–6033 10.1073/pnas.1322377111 24711410 PMC4000840

[43] Li J., Browning S., Mahal S. P., Oelschlegel A. M., and Weissmann C. (2010) Darwinian evolution of prions in cell culture. Science 327, 869–872 10.1126/science.1183218 20044542 PMC2848070

[44] Zabel M. D., and Avery A. C. (2015) Prions–not your immunologist's pathogen. PLoS Pathog. 11, e1004624 10.1371/journal.ppat.1004624 25695738 PMC4335031

[45] Orrú C. D., Hughson A. G., Groveman B. R., Campbell K. J., Anson K. J., Manca M., Kraus A., and Caughey B. (2016) Factors that improve RT-QuIC detection of prion seeding activity. Viruses 8, 140 10.3390/v8050140 PMC4885095 27223300

[46] Carreño J. M., Perez-Shibayama C., Gil-Cruz C., Printz A., Pastelin R., Isibasi A., Chariatte D., Tanoue Y., Lopez-Macias C., Gander B., and Ludewig B. (2016) PLGA-microencapsulation protects *Salmonella typhi* outer membrane proteins from acidic degradation and increases their mucosal immunogenicity. Vaccine 34, 4263–4269 10.1016/j.vaccine.2016.05.036 27372155

[47] Ertmer A., Gilch S., Yun S.-W., Flechsig E., Klebl B., Stein-Gerlach M., Klein M. A., and Schätzl H. M. (2004) The tyrosine kinase inhibitor STI571 induces cellular clearance of PrP^Sc^ in prion-infected cells. J. Biol. Chem. 279, 41918–41927 10.1074/jbc.M405652200 15247213

[48] Duque Velásquez C., Kim C., Herbst A., Daude N., Garza M. C., Wille H., Aiken J., and McKenzie D. (2015) Deer prion proteins modulate the emergence and adaptation of chronic wasting disease strains. J. Virol. 89, 12362–12373 10.1128/JVI.02010-15 26423950 PMC4665243

