# Supplementary material for: Recombinant prion protein vaccination of transgenic elk PrP mice and reindeer overcomes self-tolerance and protects mice against chronic wasting disease
Source: J Biol Chem. 2018 Nov 5;293(51):19812–22. doi: 10.1074/jbc.RA118.004810 (PMC6314114; doi:10.1074/jbc.RA118.004810)
Supplement: Supporting Information [file supp_293_51_19812__index.html]

Recombinant prion protein vaccination of transgenic elk PrP mice and reindeer overcomes self-tolerance and protects mice against chronic wasting disease — Vaccination protects against CWD — Supporting Information 

# Recombinant prion protein vaccination of transgenic elk PrP mice and reindeer overcomes self-tolerance and protects mice against chronic wasting disease

## Supporting Information

- Supporting Information (to be published online) - Supporting information
