# Supplementary material for: Recombinant prion protein vaccination of transgenic elk PrP mice and reindeer overcomes self-tolerance and protects mice against chronic wasting disease
Source: J Biol Chem. 2018 Nov 5;293(51):19812–22. doi: 10.1074/jbc.RA118.004810 (PMC6314114; doi:10.1074/jbc.RA118.004810)
Supplement: Supporting Information [file supp_RA118.004810_139334_2_supp_229228_phqfhj.pdf]

Recombinant prion protein vaccination of transgenic elk PrP mice and reindeer overcomes self-tolerance and protects mice against chronic wasting disease

Dalia H. Abdelaziz, Simrika Thapa, Jenna Brandon, Justine Maybee, Lauren Vankuppeveld, Robert McCorkell and Hermann M. Schatzl

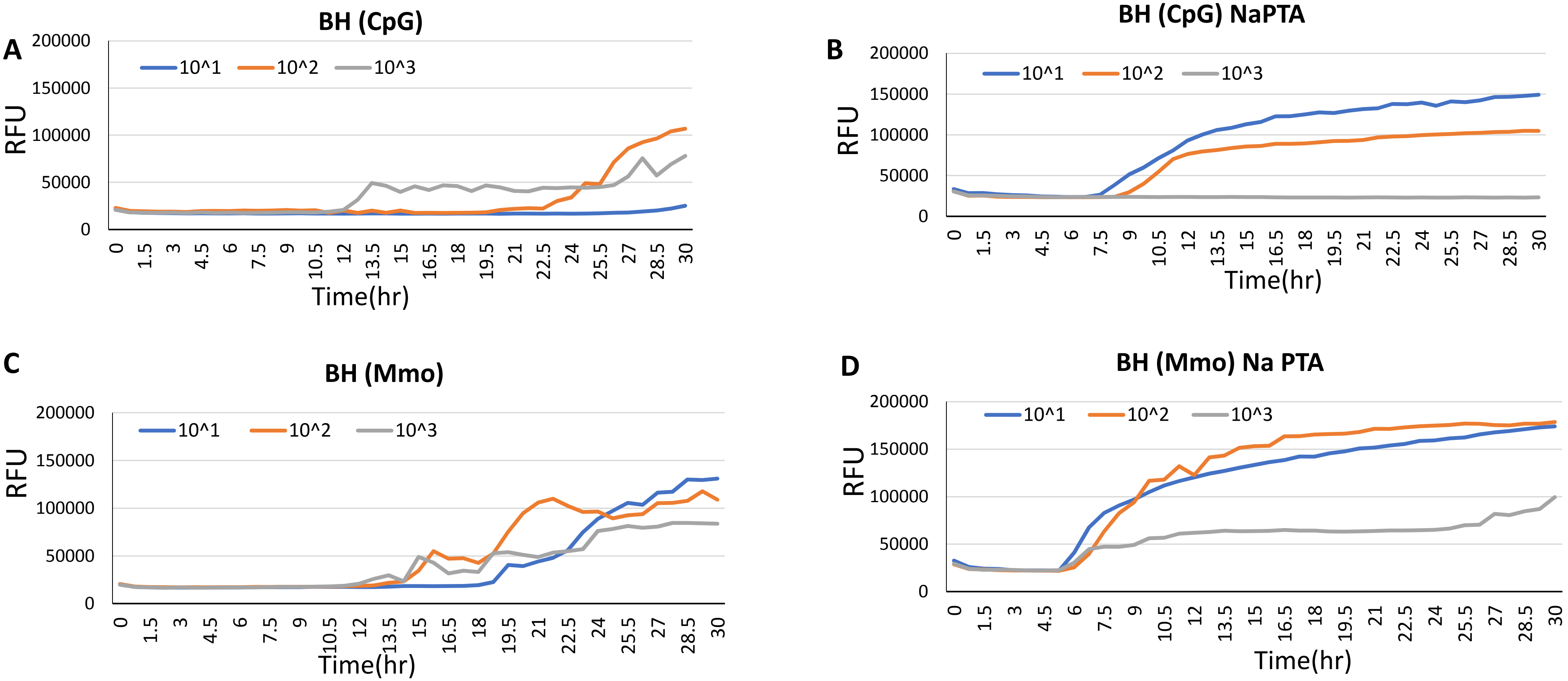

Supplementary Fig 1: Prion conversion activity in brain homogenates of intraperitoneally inoculated TgElk mice. (A) RT-QuIC expressed as relative fluorescence units (RFU) per hour in which the seed was 10% homogenate of a terminally ill CWD-infected CpG control TgElk mouse. (B) RT-QuIC in which the seed was 10% homogenate of the same mouse in panel A enriched by sodium phospho-tungstic acid (Na PTA) precipitation. (C) RT-QuIC in which the seed was 10% homogenate of a terminally ill CWD-infected Mmo-vaccinated TgElk mouse. (B) RT-QuIC in which the seed was 10% homogenate of the same mouse in panel C enriched with sodium phospho-tungstic acid (Na PTA) precipitation. Mouse rPrP was used as substrate for the reactions, three tenfold dilutions of seed were analyzed (10^{-1} to 10^{-3}), and each curve represents the average of 4 technical replicates.

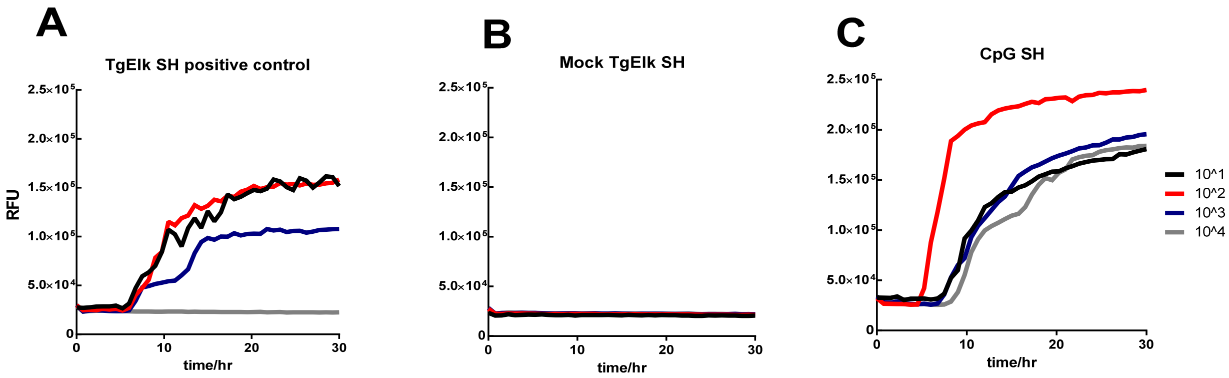

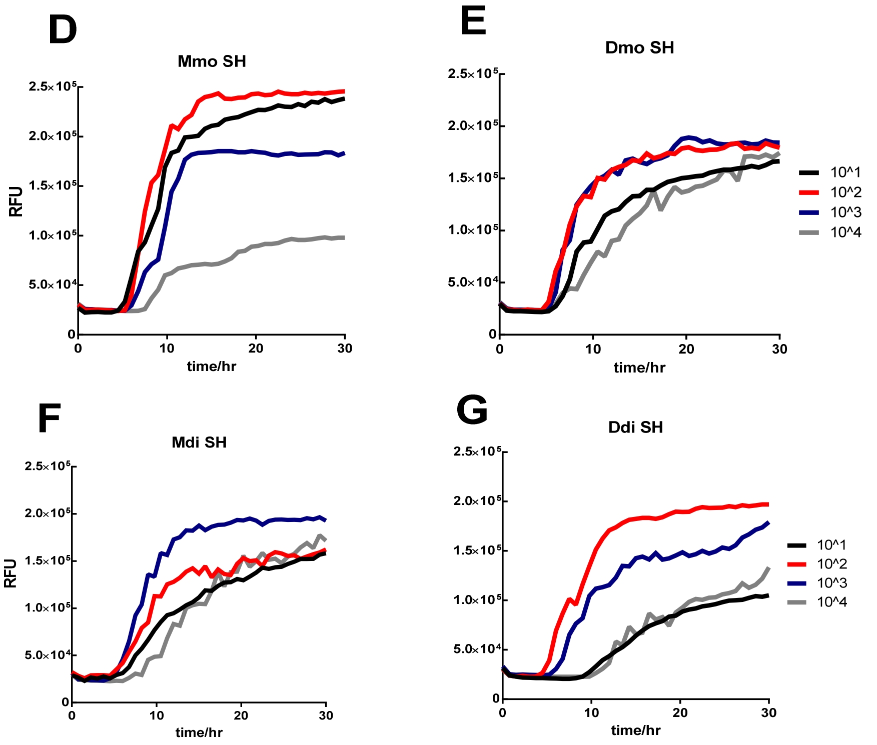

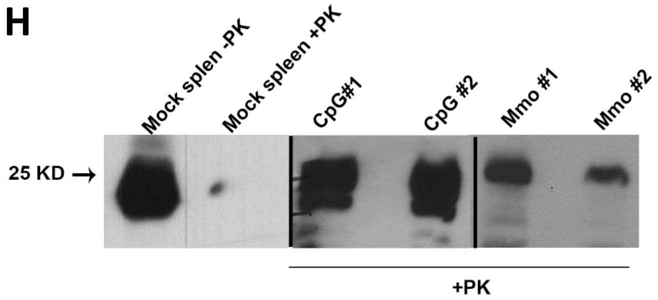

Supplementary Fig 2: Prion conversion activity and PrP^{Sc} in spleen homogenates of i.p.-inoculated TgElk mice. (A) CWD positive control for RT-QuIC in which the seed was 10% spleen homogenate of a terminally ill CWD-infected TgElk mouse. (B) Spleen homogenate of an uninfected TgElk mouse was used as negative control. (C-G) RT-QuIC assay for one representative spleen homogenate from every group. (C) CpG-only, (D) Mmo-, (E) Dmo-, (F) Mdi-, and (G) Ddi-immunized. Mouse rPrP was used as substrate for the reactions, four tenfold dilutions of seed were analyzed (10^{-1} to 10^{-4}), and each curve represents the average of 4 technical replicates. (H) Western blot analysis using mAb 8H4 of spleen homogenate samples treated with proteinase K (PK) followed by sodium phosphotungstic acid (NPTA) enrichment. Four individual mice are shown. Mock-infected TgElk spleen homogenate was used as a negative control.
